# Research on the coupling coordination and driving role of urbanization and ecological resilience in the middle and lower reaches of the Yangtze River

**DOI:** 10.7717/peerj.15869

**Published:** 2023-09-22

**Authors:** Sheng Xiao, Linghua Duo, Xiaofei Guo, Zili Zou, Yanan Li, Dongxue Zhao

**Affiliations:** 1 China University of Mining and Technology-Beijing, College of Geoscience and Surveying Engineering, Beijing, China; 2 East China University of Technology, Key Laboratory of Mine Environmental Monitoring and Improving Around Poyang Lake of Ministry of Natural Resources, Nanchang, Jiangxi, China; 3 East China University of Technology, Key Laboratory for Digital Land and Resources of Jiangxi Province, Nanchang, Jiangxi, China; 4 East China University of Technology, Faculty of Geomatics, Nanchang, Jiangxi, China; 5 Centre for Crop Science, Queensland Alliance for Agriculture and Food Innovation, University of Queensland, Brisbane, Queensland, Australia

**Keywords:** Urbanization, Ecological resilience, Resistance force, Adaptability force, Restoring force, Coupling coordination model, GTWR model, The middle and lower reaches of the Yangtze River

## Abstract

**Background:**

The growth of urbanization in the 20th and 21st centuries has resulted in unprecedented ecological security issues. The imbalance between urban development and internal ecological security is a growing concern.

**Methods:**

Based on the urban development process and the characteristics of ecosystem resilience, the corresponding urbanization evaluation system (“scale-structure-benefit”) and ecosystem resilience assessment model (“resistance-adaptability-restoring”) were constructed to explore the changes in each dimension as well as to analyze the spatial and temporal changes and driving effects of the coupled coordination level of urbanization and ecological resilience using the coupled coordination degree (CCD) model and geographically and temporally weighted regression (GTWR).

**Results:**

(1) From 2005 to 2020, urbanization levels increased (from 0.204 to 0.264, respectively), whereas the level of ecological resilience gradually decreased (from 0.435 to 0.421, respectively). The spatial distribution of urbanization is rather steady, with a “high-northeast low-southwest” pattern of regional distribution; however, the spatial distribution pattern of ecological resilience is essentially the reverse. (2) During the study period, there was an improvement in the level of coordination between urbanization and ecological resilience, with an increase from 0.524 to 0.540. However, the main coordination type remained the same, with over 46% being at the basic coordination stage. The relative development type was dominated by the lagging urbanization stage, with the lagging ecological resilience and synchronous development stages accounting for a smaller proportion, and the space was distributed in a block-like cluster. (3) The running results of the GTWR show that the core dimensions of the whole region are scale, benefit, and structure, and the impact of each dimension shows obvious spatial heterogeneity. Cities with different levels of relative development also have different central dimensions. This research will provide support for the coordination of urban development in areas where economic construction and ecological resilience are not coordinated, and will contribute to the sustainable development of urban areas.

## Introduction

Since the country’s reform and development in 1978, urbanization has increased significantly in China, with the urbanization rate of the resident population increasing from 17.9% in 1978 to 59.6% in 2018. However, while the level of urbanization is rapidly increasing, the urban ecosystem experiences disruptions and impacts that cannot be completely avoided due to multiple natural catastrophes, while ecological disorders are caused by the rough development mode of humans (Li et al., 2022; Duo et al., 2022). In the face of increasing human pressure and encroachment during urbanization, urban ecosystems urgently need to improve their resilience, that is, resistance, adaptability, and post-impact recovery, to dissipate and absorb these disturbances. In this context, achieving the coordinated development of urbanization and ecological security is not only a key issue in the world’s economic and social development but also a hot research topic in recent years. There are already a lot of study findings on the connection between urbanization and ecological resilience, but there are still numerous difficulties that demand the academic community’s full attention. Firstly, there is a great deal of spatiotemporal variability since different cities have developed in different ways, leading to distinct characteristics in each region’s urbanization process and urban ecological resilience. Therefore, it has some practical value to build a targeted evaluation system based on the real circumstances of the study topic. Secondly, since the impact of urbanization and ecological resilience is not homogeneous, and current research frequently ignores the spatiotemporal non-stationary nature, it is necessary to investigate the nonlinear relationship between urbanization and ecological resilience through the geographically and temporally weighted regression (GTWR) model and analyze the local differences in driving factors. To fill this gap, this study has constructed corresponding urbanization and ecological resilience assessment systems, to explore the changes in each dimension, and to analyze the spatial and temporal changes and driving effects of the coupled coordination level of urbanization and ecological resilience to provide a policy basis for the coordinated development of urban areas.

Resilience, which means “rejuvenation,” was first used in the field of systems ecology to gauge how resilient ecosystems are to stress changes (Holling, 1973; Meerow, Newell & Stults, 2016; Mayar, Carmichael & Shen, 2022). With continuous domestic and international research, the concept of “resilience” has been expanded beyond the field of natural ecology to a variety of fields (Li, 2017; Angeler & Allen, 2016; Walker, 2020). As the main space for human activities, “urban resilience” naturally comes into the view of scholars (Folke, 2006). Although scholars have not yet agreed on a single meaning for the term “urban resilience,” the majority of them agree that it refers to the capacity of cities to withstand, adapt to, and recover rapidly from external forces and risks (Xu et al., 2018; Song et al., 2019), which includes social, economic, ecological and infrastructural resilience, among dimensions (Zhao, Fang & Liu, 2020). As one of the key dimensions of urban resilience, the assessment and analysis of ecological resilience has been closely studied by many scholars. Existing research focuses on the measurement and analysis of the relationship between urbanization and the ecological environment, and scholars have mostly analyzed the coupling relationship between urbanization and the ecological environment based on the system theory paradigm, using various methodological models such as the coupling coordination model (Chen et al., 2020; Liu et al., 2018a), Environmental Kuznets Curve (EKC) (Xu, Dong & Yang, 2018), LMDI method (Ding & Li, 2017), gray correlation (Zhang et al., 2017), and geographically weighted regression (Liang, Wang & Li, 2019). For example, the quantitative evolution of the relationship between urbanization and ecological environment development has been analyzed using the coupled coordination degree model (Fu et al., 2020). The EKC model, which is based on economic urbanization and ecological environment endowment variables, enhances the coupling analysis of urbanization and ecological environment in the Yangtze River Delta of China (Zhao, Wang & Zhou, 2016). Exploratory spatial data analysis is used to study the spatial pattern and clustering of the coupling and coordination of new urbanization and ecological stress systems (Yao et al., 2019). In addition, an increasing number of studies have focused on the dynamic changes in the coupling relationship between urbanization and the ecological environment, and have attempted to adopt SD, CA, artificial intelligence, and other technical methods to simulate the dynamics of the coupling relationship (Wei et al., 2017; Wan et al., 2017; Fang et al., 2020).

At the same time, ecological resilience and urbanization have been studied by other academics, and studies have argued that ecological resilience is destroyed as a result of the urbanization process’s increase in land use (Chen & Yuan, 2017). The hindering impact of urban growth on ecological resilience is enhanced due to the imperfect ecological protection mechanism (Chen & Xia, 2020), and others construct the “scale-density-shape” three-dimensional ecological resilience evaluation system to assess the coupled coordination degree (CCD) (Wang et al., 2021). However, the level of resilience varies between urban development stages, with ecological resilience declining during the early stages of expansion as the city grows, ecological risk peaking, and urban ecological functions gradually recover (Li, Sun & Zheng, 2021).

In conclusion, research on the interaction between urbanization and ecology has advanced, but from the perspective of resilience characteristics, there are few studies on the driving analysis of the coordination and coupling between urbanization and ecological resilience, failing to further explore the driving roles and differences of each factor, making it challenging to link the research findings with urban spatial locations to provide more scientific guidance in urban planning.

Regression analysis is a common method to explore the influencing factors. Although the traditional linear regression analysis can analyze whether the positive and negative correlations of the influencing factors are significant, it is unable to accurately identify the importance of the factors because of multicollinearity, bias, and other problems. In recent years, more advanced regression models such as machine learning and random forest have overcome the impact of multicollinearity and can assess the importance of the influencing factors, it has an ideal fitting effect and is widely used in ecological environments, hydrology, epidemiology, and other fields, but it does not fully consider the spatial heterogeneity and time lag effect of various influencing factors. The spatiotemporal geographic weighted regression (GTWR) model can conduct research from different spatiotemporal dimensions, analyze the intensity and direction of driving factors in each research unit, grasp the root causes of spatiotemporal differentiation of research objects, and provide an important basis for regional ecological zoning management and the formulation of targeted ecological protection policies.

To address these shortcomings, this study constructs a quantitative measurement system of urbanization and ecological resilience, assesses the CCD of urbanization and ecological resilience through a panel data study of 77 cities, and reveals the spatial and temporal characteristics and core driving factors of the coupled coordination level of the two. The core driving factors were then identified based on the relative development levels of urbanization and ecological resilience to provide targeted countermeasures for different types of cities. The findings of this study can help the government better understand the complex coupling relationships and formulate sustainable urbanization development policies to balance urbanization and ecological security.

## Materials & Methods

### Study area

The middle and lower regions of the Yangtze River are crucial components of the Yangtze River Economic Belt, spanning six provinces and cities, including Shanghai, Jiangsu, and Jiangxi, among others, including the Wuhan City Circle, Changzhutan City Cluster, and Poyang Lake City Cluster. This is essential for China’s regional development plan (Liu et al., 2020; Chen et al., 2020). The Yangtze River Economic Zone, which is responsible for regulating the climate, removing pollutants, and storing floods, also plays a vital ecological role. However, the pursuit of a high economic level and disregard for the “quality” development of urbanization has led to increasing pressure on the source environment of the middle and lower reaches of the Yangtze River and the prominence of ecological and environmental problems (Zhang et al., 2021; Yang et al., 2021; Bai et al., 2019). This makes it appropriate to study the coupled and coordinated development relationship between urbanization and ecological resilience.

**Figure 1:**
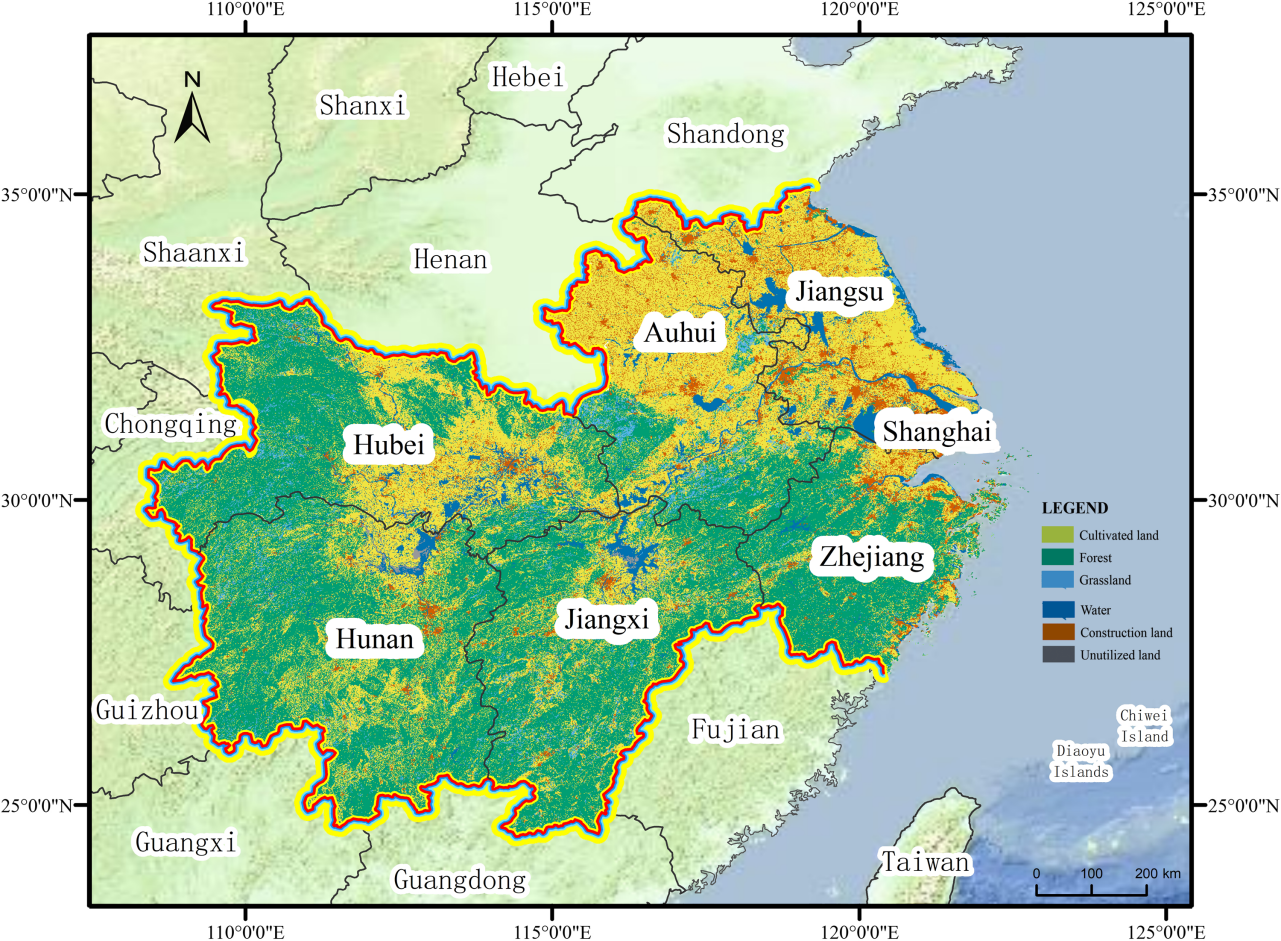
Study area.

Therefore, considering the cities in the middle and lower reaches of the Yangtze River as the research area, the remaining 77 cities at the prefecture level and above were analyzed and evaluated as the study unit, considering that five cities, including Xiantao, Tianmen, and Qianjiang, significantly changed their administrative boundaries during the study period, as shown in Fig. 1.

### Data sources

The data types displayed in this research include raster, vector, and statistical data, with the spatial data of various resolutions uniformly resampled to 1,000 m × 1,000 m. Specifically: 1,000 m × 1,000 m resolution land use maps from 2005 to 2020 from the Resource and Environmental Science and Data Center and Earth System Science Data (https://figshare.com/articles/dataset/Land_use_raster_data_zip/21707603); statistical data on urbanization indicators such as per capita GDP, fixed asset investment and unit employment were from the statistical annual report of each province and the “China Urban Statistical Annual report” (https://figshare.com/articles/dataset/China_Urban_Statistical_Annual_report_zip/21707606); The percentage of non-agricultural land were gathered from the data on land use from remote sensing (https://figshare.com/articles/dataset/Percentage_of_non-agricultural_land_of_cities_from_2005_to_2020/21731015). In addition, the trend extrapolation or interpolation method was used to process missing data to ensure the completeness and accuracy of the data.

### Methods

**Figure 2:**
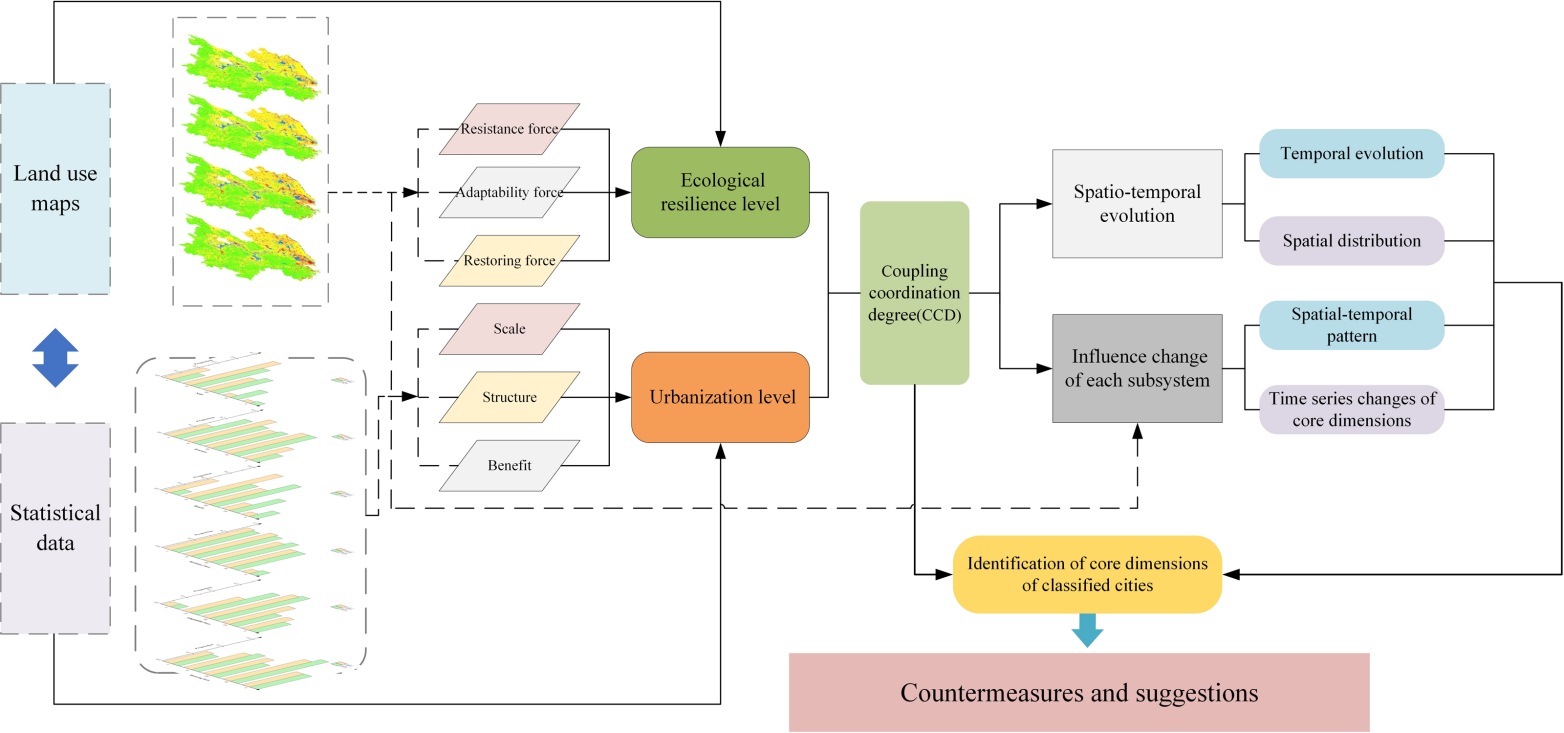
Research framework of this article.

The research presented in this article can be divided into three sections (Fig. 2). Firstly, an evaluation model for urbanization and ecological resilience was established according to the urbanization process and resilience characteristics. Following the standardization of the raw data, the AHP-TOPSIS method was used to calculate the comprehensive value. Secondly, the spatiotemporal changes were visually evaluated once the CCD between the two was calculated using the coupling coordination model. Finally, to provide appropriate countermeasures and recommendations, the influence of each dimension is examined based on the GTWR model, the temporal and spatial changes of regression coefficients of each dimension are summarized, and the core dimensions of various types of cities are identified.

### Indicator system of urbanization and ecological resilience

Urbanization is an integrated process that reflects not only the growth of the urban population but also the expansion of urban land, culture, and lifestyle into rural areas (Weng, 2007; Bekhet & Othman, 2017). The rural economy is experiencing a shift from traditional agriculture to secondary and tertiary industries as well as from decentralized family operations to agglomeration-based production, with significant changes in population, land use, industry, and employment. Based on the indicators of the above-mentioned main change dimensions and considering the accessibility of indicators in the study area, this study constructs a comprehensive indicator system based on “scale-structure-benefit”. The urbanization index system was constructed with reference to previous work (Liu et al., 2021; He et al., 2017; Zhang, Wei & Ding, 2020), and the most cited and comprehensive basic indicators were collected to reflect the level of the urbanization process. Thus, the urbanization index system consists of three main dimensions and 15 basic indicators (Table S1).

Based on the environmental pressure-state-response framework, which is widely applied to ecological environments (Hughey et al., 2004; Zheng et al., 2020), an ecological resilience evaluation system containing three first-level indicators (resistance, adaptability, and restoration) was established (Table S2). Through this idea, the ecological resilience of cities is assessed as the result of the joint influence of three factors: pressure risk, current conditions, and spatial and temporal responses. First, resistance to pressure in terms of natural disaster potential and ecological degradation risk is brought about by human activity. Many studies have shown that ecosystem resistance is closely related to ecosystem service functions. Then the ability of how quickly the existing state of the city adapts when facing the pressure to minimize its negative impact is considered as the corresponding main current state of the city. The more stable an ecosystem, the more adaptive it is. Finally, the ability of the urban ecosystem to recover quickly to its original state after suffering the aforementioned adverse impacts is considered to be its spatial and temporal response.

Due to differences in the order of magnitude, dimension, and positive and negative effects of urbanization and ecological resilience indicators, the original data were uniformly standardized.

Positive indicators:

(1) \begin{eqnarray*}{\mathrm{x}}_{ij}= \frac{{X}_{ij}-\min \nolimits ~{X}_{ij}}{\max \nolimits ~{X}_{ij}-\min \nolimits ~{X}_{ij}} \end{eqnarray*}

Negative indicators:

(2) \begin{eqnarray*}{\mathrm{x}}_{ij}= \frac{\max \nolimits ~{X}_{ij}-{X}_{ij}}{\max \nolimits ~{X}_{ij}-\min \nolimits ~{X}_{ij}} \end{eqnarray*}

where x_*ij*_ is the standardized data, X_*ij*_ is the original data, and max X_*ij*_ and min X_*ij*_ are the maximum and minimum values of the original data, respectively.

After standardizing the original data and assigning weights using the AHP and entropy method (Table S3), it was found that the weights of the indicators calculated by the entropy method were not consistent with reality because of the large number of study units and large internal differences of individual indicators. To reflect the scientific and fairness of indicator assignment, this study used AHP to establish weights. At the same time, because the comprehensive evaluation of urbanization and ecological resilience involves multiple dimensions and the TOPSIS method is often used to solve multi-attribute decision problems, relying on the actual objective data of the study area, the AHP-TOPSIS model is more suitable for the evaluation of this study. The calculation process is not repeated here because the method is more general.

### Coupling coordination relative development model

The model is introduced to reflect the coupling coordination degree (CCD) between urbanization and ecological resilience levels. The relative development coefficient, E, was introduced to evaluate the relative development status of the two (He et al., 2017; Liu, Zhang & Zheng, 2011).

(3) \begin{eqnarray*}C& ={ \left\{ {\mathrm{u}}_{1i}\ast {u}_{2i}/{ \left[ \left( {u}_{1i}+{u}_{2i} \right) /2 \right] }^{2} \right\} }^{1/2}\end{eqnarray*}

(4) \begin{eqnarray*}D& =\sqrt{C\mathrm{ \ast }T}\end{eqnarray*}

(5) \begin{eqnarray*}T& =x{u}_{1i}+y{u}_{2i}\end{eqnarray*}

(6) \begin{eqnarray*}E& ={u}_{1i}/{u}_{2i}\end{eqnarray*}

In the formulas, C represents the coupling degree; *μ*_1i_ and *μ*_2i_represent the comprehensive score of urbanization and ecological resilience of city *i*; D is the CCD; T stands for the total coordination index of the two subsystems of urbanization and ecological resilience; and *χ* and *γ* represent the importance degree. In this study, considering that urbanization and ecological resilience are equally important (Liu et al., 2018b; Li & Yi, 2020), *χ* and *γ* are taken as 0.5. Based on related studies (Liu et al., 2018a; Liu et al., 2018b; Wang et al., 2019), D is divided into six types: 0.2, 0.4, 0.5, 0.6, and 0.8 as the dividing points. E denotes the relative development degree of urbanization level and ecological resilience level when 0<E ≤0.8, indicating that the level of urbanization lags, when0.8<E<1.2, indicating that the two develop simultaneously; and when *E* ≥ 1.2, indicating that the ecological resilience lags.

### GTWR model

The GTWR model, which can estimate the factor parameters more accurately, combines spatial heterogeneity and temporal non-smoothness coupling into the GWR model. This model allows the change in the coupled coordination level of urbanization and ecological resilience to be seen as a full spatiotemporal evolutionary process to more thoroughly study the driving mechanism of the coupled coordination level of urbanization and ecological resilience (Huang, Wu & Barry, 2010; Fotheringham, Crespo & Yao, 2015).

(7) \begin{eqnarray*}{y}_{i}={\beta }_{0} \left( {u}_{i},{v}_{i},{t}_{i} \right) +\sum _{1}^{k}{\beta }_{k} \left( {u}_{i},{v}_{i},{t}_{i} \right) {x}_{ik}+{}_{i},i=1,2,\ldots ,n\end{eqnarray*}

where *y*_*i*_ is the observed value, (*u*_*i*_, *v*_*i*_, *t*_*i*_) is the spatio-temporal coordinate of the *ith* sample point, *β*_0_ (*u*_*i*_, *v*_*i*_, *t*_*i*_) is the regression constant at point *i*, *β*_*k*_ ( *u*_*i*_, *v*_*i*_, *t*_*i*_) is the *kth* regression parameter at point *i*, *x*_*ik*_ is the value of the independent variable *x*_*k*_ at point *i*, and *ɛ*_*i*_ is the independent random error term.

## Results

### Analysis of urbanization level

#### Analysis of scale level

The average values of the scale levels from 2005 to 2020 are 0.1239, 0.1485, 0.1688, and 0.1574, respectively, showing a rising and then declining trend, indicating that with the advancement of the national economic construction and rapid improvement of socioeconomic level, the level of urban scale has been increasing, especially the rapid expansion from 2005 to 2015 brought substantial urban socio-economic growth.

**Figure 3:**
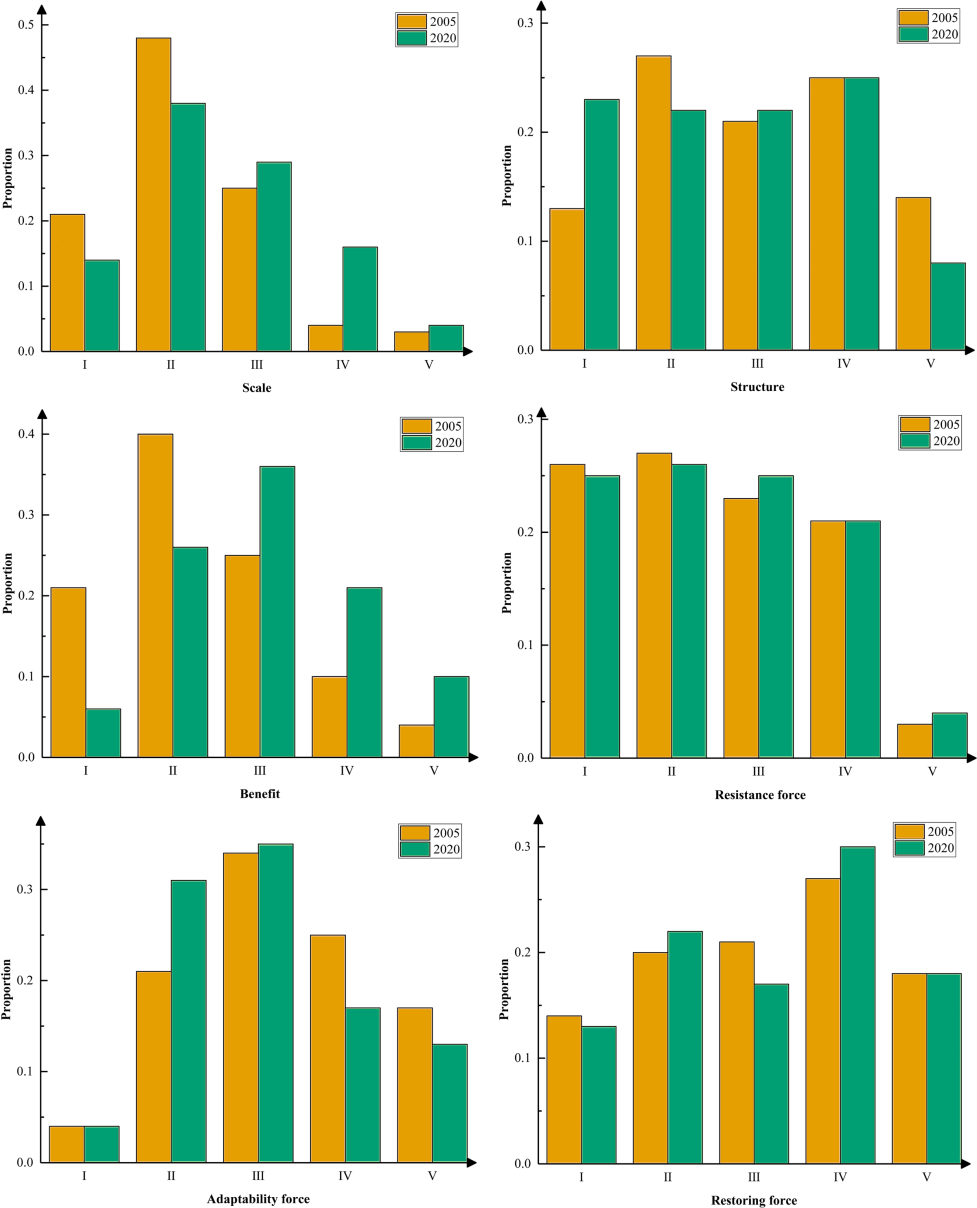
The change of all dimension index, 2005–2020.

The natural breakpoint method divides the scale level into five levels (I–V): low, relatively low, medium, relatively high, and high. This allowed for a more thorough investigation of the characteristics of changes in the scale. The cities of each type were counted along with their percentages (Fig. 3). The results show that the level of urban scale from 2005 to 2020 is mainly dominated by low, relatively low-level, and medium-level urban classes. More than 80% of the total is composed of three. At the beginning of the study period, the level of urban scale was dominated by low levels. Towards the end of the study period, each city’s scale level typically increased due to construction and development, and the share of medium- and high-level cities gradually increased. The proportion of high-level cities is more consistent and less variable. At the relatively high level, the number of cities increased significantly by 12%. The medium-level urban class also showed a slight increasing trend, rising from 25% in 2005 to 29% in 2020. Low and relatively low-level city classes showed a decreasing trend, from 21% and 48% in 2005 to 14% and 38% in 2020, respectively. The variations are substantial, with a 10% decrease in the relatively low-level city classes being the most noticeable. The scale of cities in the study area is generally increasing, with medium, low, and relatively low-level classes of cities moving up to higher classes. This is primarily due to the nation’s rapid expansion and implementation of several economic policies during the research period. Urbanization has accelerated substantially, leading to considerable increases in built-up areas, population density, and industrial production.

#### Analysis of structure level

Throughout the study period, the mean urban structure level decreased from 0.409 to 0.382. Urban growth and buildings consume and use a variety of resources inefficiently. Consequently, the structural layout of urban land space, industry, and population changes rapidly. The level of the urban structure eventually declines. Figure 3 shows the variations in the structure level classes between 2005 and 2020. The share of cities in the low-level class continued to rise by 10%. The percentages of medium- and relatively high-level cities were comparatively constant and did not change significantly. The relatively low-level and high-level cities shrank slightly, with decreases of 5% and 6%, respectively. It can be seen that the change in the level of urban structure from 2005 to 2020 is different from the change in the level of scale, showing a general downward trend. Medium and high levels are converted to low levels. It also shows how the city’s consumption and usage of various resources and space have expanded as a result of the socioeconomic building and development of the metropolis. The layout of various resource utilization structures has an impact on the creation of high-quality urban development; therefore, it is vital to optimize the level of urban structures.

#### Analysis of benefit level

The average city benefit level increased from 0.219 to 0.341 between 2005 and 2020, showing an overall upward trend. This indicates that the level of urban effectiveness is rising annually. The figure shows that from 2005 to 2020, the share of cities with medium, high, and relatively high levels of urban efficiency will increase. Grades at the middle and high levels also increased by 11%. The overall percentage of relatively low and low-level class cities showed a continuous decreasing trend and considerable changes during the study period, with a decrease of at least 14%. Simultaneously, low and relatively low-level cities, which accounted for more than 60% of the total at the beginning of the study, predominated and played a decisive role in the overall level of city effectiveness. In contrast, the two levels combined accounted for 32% of the total in 2020, which has little effect on the level of urban effectiveness as a whole and is highly variable. Overall, the main characteristics of the city’s benefit level during the study period were the continuous decline in the share of relatively low and low-level cities, as well as the yearly increase in the share of medium and relatively high levels. This indicates a general increase in city benefits across the study period, with varying levels of change and variation across cities.

**Figure 4:**
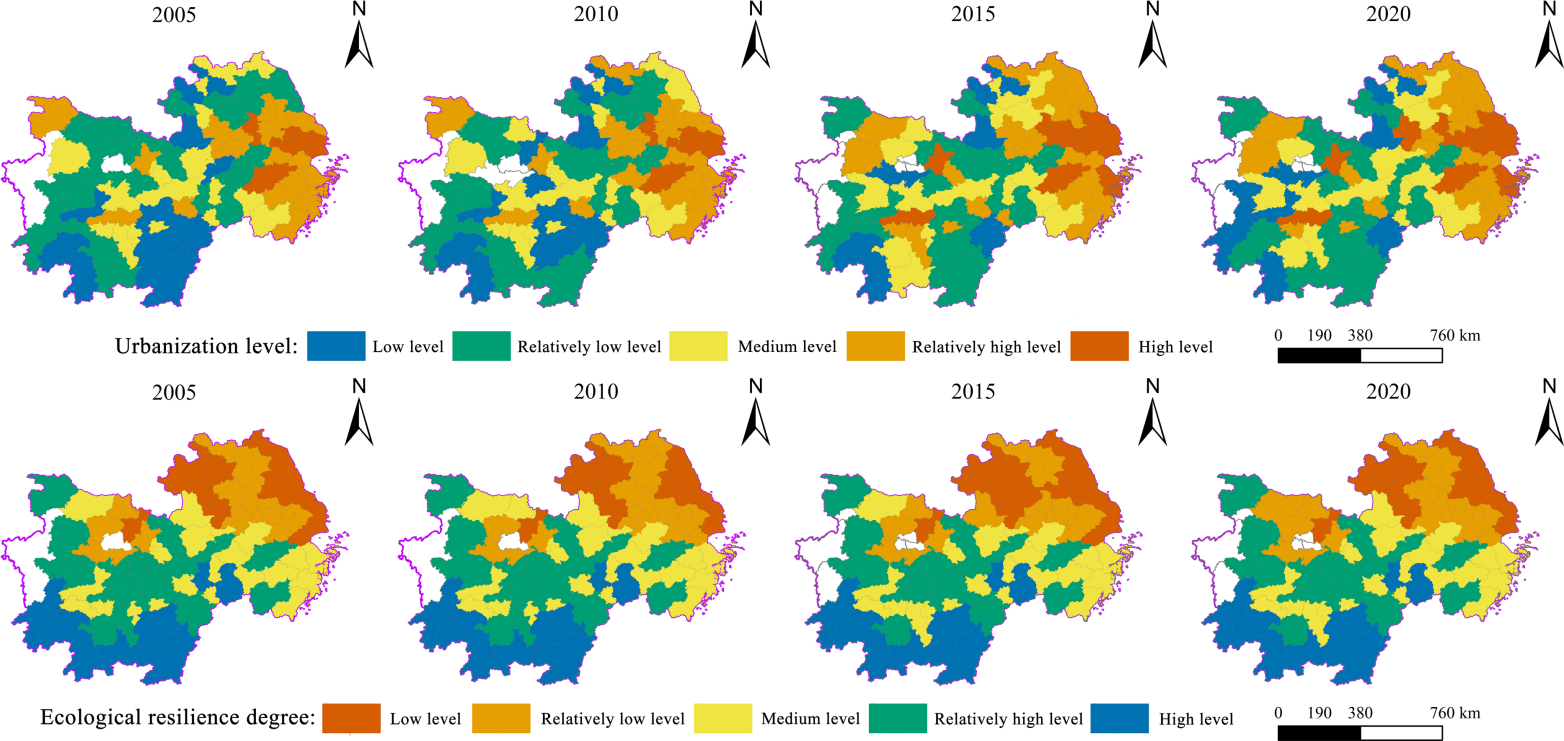
Spatial distribution of urbanization and ecological resilience level of cities, 2005–2020.

Based on the “scale-structure-benefit” evaluation system constructed above, the AHP-TOPSIS method was used to measure the comprehensive urbanization level of cities. The results showed that the average urbanization levels from 2005 to 2020 were 0.204, 0.205, 0.253, and 0.264. In terms of spatial distribution, the overall urbanization level from 2005 to 2020 demonstrates a clear spatial distribution pattern of “high in northeast and low in southwest.” Spatial divergence features were significant (Fig. 4). Low and relatively low-level class cities occupy a larger section of the study area, mostly in the southwestern region. For example, cities in the Jiangxi, Hunan, and Hubei domains were distributed in a belt-like cluster. The major cities of each province are graded as being at a relatively high level, which is closely related to the degree of urban economic development. The majority of high and relatively high-level cities, including Shanghai and Hangzhou, are located in the eastern and northeastern portions of the study area. The cities in the medium and high levels of the hierarchy gradually spread and expanded to the periphery, while the relatively low levels shrank significantly, as shown in the figure. High-level regional expansion is a tendency in the east, whereas low-level regional contraction is a trend in the west. Additionally, the areas surrounding Wuhan, Changsha, Nanchang, and Hangzhou are particularly representative of this upward movement in cities.

### Analysis of ecological resilience level

#### Resistance force

The average resistance force levels from 2005 to 2020 were 0.2399, 0.2395, 0.2401, and 0.2417, respectively, showing decreasing and then increasing trends. This demonstrates that as the nation’s economy grows, the scale of construction land increases, and woodlands, waters, and other land types shrink, which has a negative impact on ecosystem service values and increases vulnerability. As the country pays attention to the ecological environment at a later stage, ecological lands such as forests and grasslands are protected and restored. The value of ecosystem services has improved and urban ecosystem resistance has also increased. To thoroughly examine how the resistance force level fluctuated, the number and proportion of cities of each type were estimated (Fig. 3). The results show that the level of resistance force in 2005–2020 has the lowest percentage of high-level classes at 3% and 4%, respectively. The other four levels accounted for a more evenly percentage, all above 20%. Among them, low and relatively low levels are the main ones, and their sum is above 50%, while high and relatively high levels make up a slightly lower percentage. The level of resistance force at the beginning of the study period was dominated by low and medium levels, and the level of resistance force generally increased across cities in 2020 as a result of the comprehensive development of urban areas. The percentage of medium to high-level cities has increased annually, with limited growth. In general, the level of urban resistance force showed an increasing trend; cities with medium, relatively low, and low levels were all converted to higher levels. This was mostly due to the promotion of various sustainable development concepts during the study period, which gradually increased the emphasis on urban ecological construction in different locations. The level of the urban resistance force improved but only slightly.

#### Adaptability force

The average urban adaptability force levels from 2005 to 2020 were 0.5697, 0.5729, 0.5311, and 0.5249, respectively, indicating a tendency of growth and subsequently decline. This is primarily because attributes such as landscape fragmentation and diversity dictate the level of the adaptation force of urban ecosystems. With the development of urban economic construction from 2005 to 2010, the economy and nature developed together and the level of adaptability force improved significantly. Due to the excessive pursuit of social and economic construction from 2010 to 2020, ecological land, such as forest land, grassland, and water area, has shrunk to varying degrees. The landscape has become more fragmented and homogenized, which has decreased its adaptability. The figure shows that the percentage of cities with medium and relatively low levels showed an increasing trend during the study period. The low levels of the increase reached 10%. The share of high and relatively high-level cities demonstrated a decreasing tendency during the course of the study period, and the trend was more noticeable. The study period was dominated by the highest percentage of medium-level cities (both over 34%), which played a decisive role in the level of adaptability force as a whole. The continuous increase in the share of low-level cities and the yearly decrease in the share of high and relatively high levels. This indicates a general trend of decreasing adaptability force levels during the study period, although the difference varies between cities.

#### Restoring force

The mean urban restoring force levels during the study period were 0.5183, 0.5180, 0.5192, and 0.5192, respectively, showing an overall increasing trend. This indicates a general trend of increasing restoring force levels across cities during the study period. Figure 3 shows that the changes in the different levels varied from 2005 to 2020. Among them, the low- and high-level cities were more stable. The percentage of medium-level cities decreased slightly. Cities with the relatively low and relatively high-level cities expanded slightly, with increases of 2% and 3%, respectively. It is clear that there is a slight-rising tendency in the restoration level change from 2005 to 2020. This shows that, with the construction and development of the urban economy, the rate of land resource utilization gradually tends to stabilize. Although the variation in the overall land-use structure was minor, there were some variations between different cities.

The cities’ mean ecological resilience levels from 2005 to 2020 were 0.4350, 0.4361, 0.4228, and 0.4210, respectively, with a general decreasing trend. In terms of its spatial distribution, the overall ecological resilience level from 2005 to 2020 shows a stable spatial distribution pattern of “high in southwest and low in northeast,” with significant spatial divergence (Fig. 4). High and relatively high-level cities, such as those in Jiangxi, Hunan, and Hubei, are distributed in a belt-like cluster and occupy a specific amount of space, mostly in the southwest of the research region. The central cities of the provinces at a medium level, which is closely linked to their natural environmental conditions. The low-and relatively low-level cities were mainly located in the eastern and northeastern regions of the study area, and the majority of them were block-like clusters. The low-level classes of cities gradually expanded, as seen by the spatial distribution of ecological resilience levels in the four-time cross-sections. For instance, the Hubei region’s dispersion of agglomerations was concentrated in Xiaogan. The relatively high levels have reduced slightly, similar to the band-distributed medium-level region in Hunan Province, with Yiyang as its center of expansion.

### Coupling analysis of urbanization and ecological resilience

**Figure 5:**
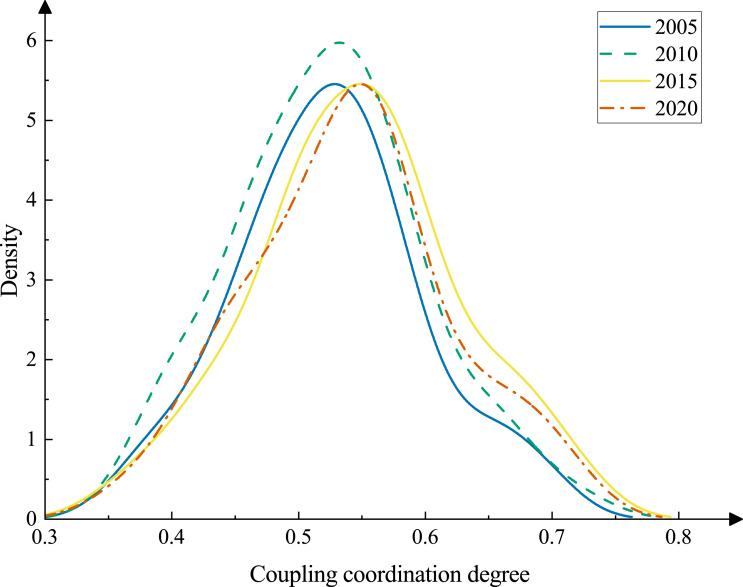
The evolution of CCD of urbanization and ecological resilience, 2005–2020.

To reveal the time-series evolution characteristics of the CCD, the years 2005, 2010, 2015, and 2020 were selected for the kernel density estimation analysis (Fig. 5). As the figure illustrates, the kernel density curve position shows a general rightward shift from 2005 to 2020. Among them, the overall rightward trend of the curve is more obvious between 2010 and 2015, and this phenomenon reflects that the overall CCD level shows a trend of slow but steady growth. The left and right ends of the curve indicate the existence of two levels of differentiation. A certain number of cities gather at high and low values, and there are clear trailing features that point to the presence of two levels of differentiation. It is clear that the low-value cities shrink slightly and the high-value cities gradually increase. On the change of the peak, the peak of the wave narrows slightly and the peak of the curve shows a decreasing trend, which indicates that the main type of CCD in the study area remains unchanged, the relative difference between cities increases and the overall level improves.

**Figure 6:**
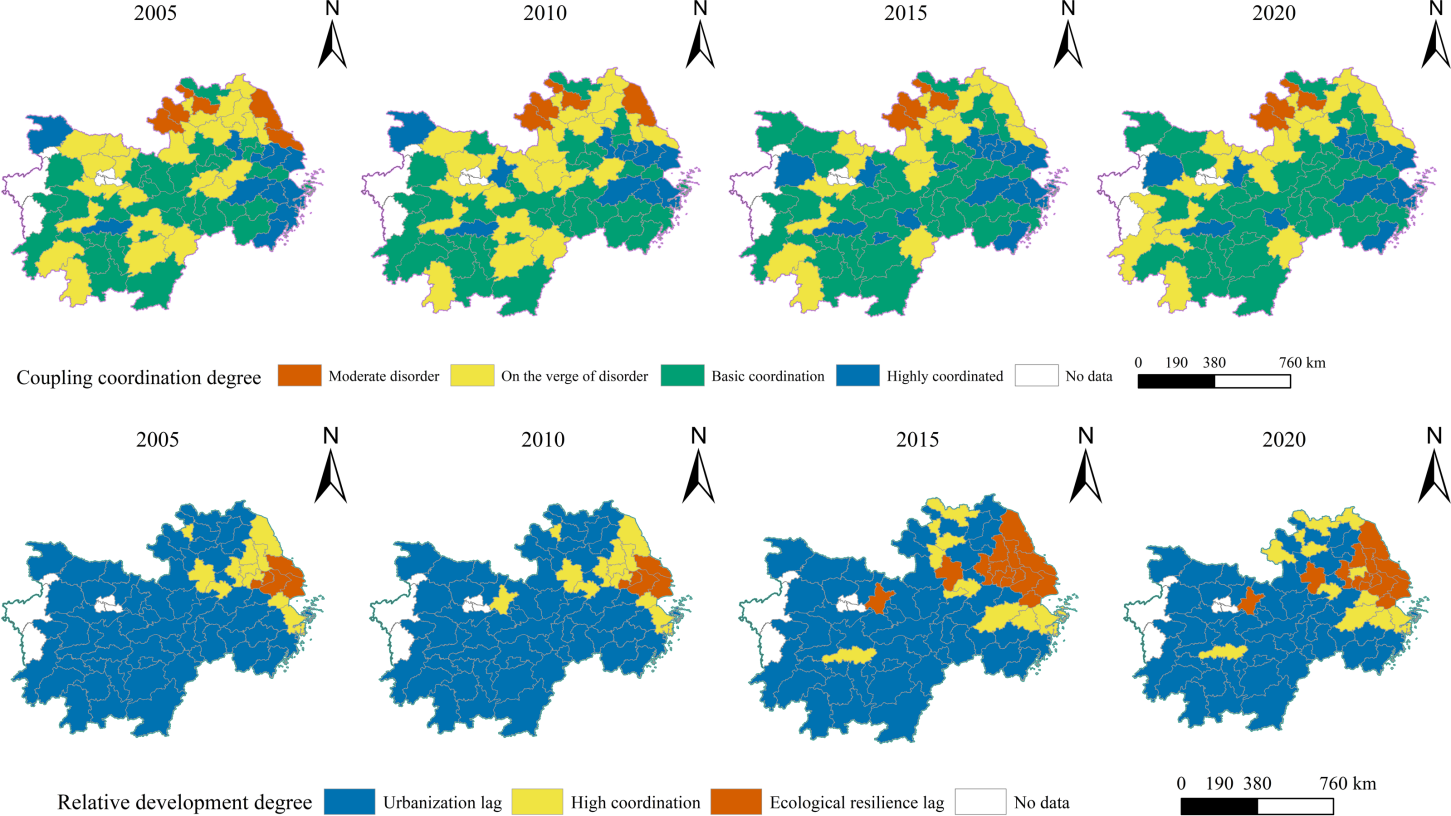
Spatial distribution of CCD of and relative development degree, 2005–2020.

As shown in Fig. 6, cities at the level of good coupling coordination stage expand year by year and are primarily distributed in the eastern coastal cities of the study area, such as Shanghai, Ningbo, Shaoxing, and Hangzhou. The overall increase in stage at the end of the study period was limited. Cities in the basic coordination stage, which are mainly transformed from cities in the near-disorder stage, comprise the majority of the cities in the study area. Its expansion trend is obvious and extensive, spreading from Jiangxi and Zhejiang to the Hunan and Hubei regions, with a belt and block-like clustering distribution. The near and moderate disorders show a shrinking trend, mainly distributed in Anhui and Hubei. Among them, moderate disorder turns into near disorder and near disorder turns into basic coordination, and the trend of upward transformation is obvious. The degree of CCD of urbanization and ecological resilience in each city exhibits an upward trend. The stage that makes up the majority remains unchanged, and the changes in the stages of good coupling coordination and moderate disorder are limited.

 The urban distribution pattern was more stable in terms of the spatial distribution of the relative degree of development. The lagging urbanization stage is the main stage and is mainly distributed in Jiangxi, Hunan, Hubei, and Anhui. Synchronous development and ecological resilience lag stages were primarily found in the northeastern coastal cities of the research area. Cities in the lagging stage of ecological resilience were more variable, mostly centered on Shanghai and Suzhou, which gradually spread to the periphery and were distributed in a block-like cluster. Individual provincial capitals, including Wuhan and Hefei, were analogous to the former. In comparison, the fluctuation of cities in the synchronous development stage changes less. Overall, it is evident that cities in the lagging stages of urbanization predominated during the study period. Some cities have been transformed into synchronous development areas owing to improvements in their economic and environmental conditions. Therefore, the ecological resilience level should not be ignored.

### Analysis of influence change of each subsystem

The values for each dimension of the cities were obtained, and the extreme difference standardization method was used to eliminate the magnitude of each dimension value. With the values of the six dimensions as independent variables and the CCD between urbanization and ecological resilience as dependent variables, the influence coefficients of each dimension in the model were determined using Huang’s spatiotemporal geographically weighted regression plug-in. Among the model parameters, the R2 of the GTWR model and the corrected R2 were higher than 98%, the AICc was −1,735.01, and the residual squares and Sigma values are smaller (Table S4).

### Spatial and temporal pattern analysis of regression coefficients in all dimensions

**Figure 7:**
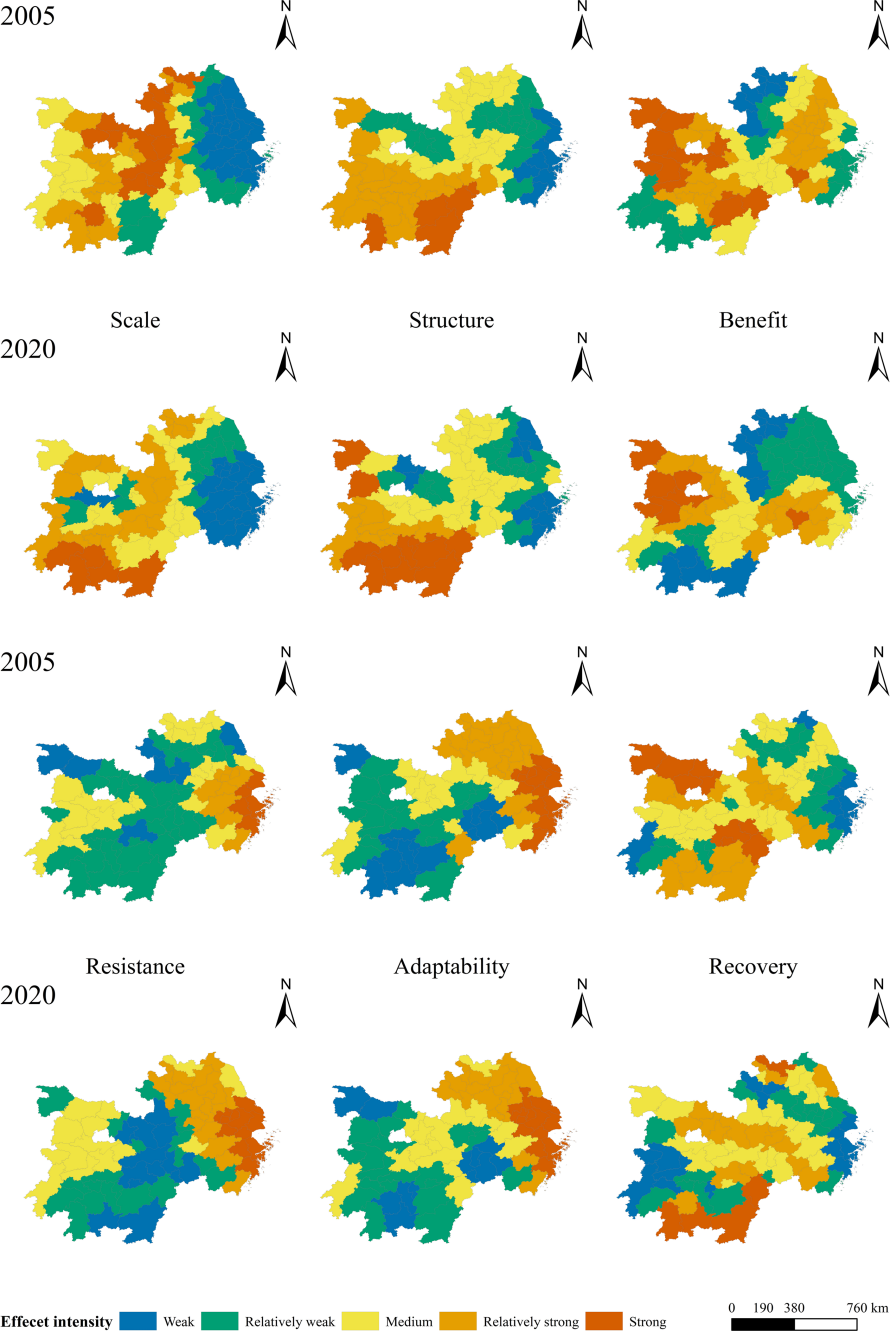
Spatial pattern of regression coefficients of all dimensions.

Figure 7 shows that the high-intensity area of the scale dimension from 2005 to 2020 has shifted and expanded from the central part of the study area to the southwest, while the low-intensity area is primarily found in the eastern coastal region, and its range is continuously contracted. The scale dimension shows a general increase in the level of intensity as well as a conversion of the low-level range to a high level and a growth at each intensity level. The majority of the 2005 strong levels were distributed in Nanchang, Jingdezhen, and other cities that border the provinces of Hubei, Anhui, and Jiangxi. Most cities in Hubei, Hunan, and Jiangxi will be of strong or relatively strong grades by 2020, while the range of weak grades in the east shrinks significantly and is distributed in Shanghai and Zhejiang, forming a bifurcation between the east and west.

 From 2005 to 2020, Jiangxi and southern Hunan Province were the main locations of the relatively strong impact areas in the structural dimension, with a gradual expansion trend and belt-like distribution. The relatively strong impact areas in 2005 were located in Ganzhou, Ji’an, and Yongzhou, and expanded to the west along the boundary of the study area in 2020, while the overall change in the weaker impact areas was not significant and located in the cities of Shanghai, Jiangsu, and Zhejiang area cities. Cities are currently developing in a high-quality manner, focusing on the adjustment and optimization of economic structure, prompting land use changes and efficiency improvements. The economic, land use, and population structure of cities affect the comprehensive coordinated level; therefore, the structural layout of slow-developing cities in central Jiangxi and Hunan needs to be further optimized to promote sustainable urban development.

The spatial pattern of the change in the benefit dimension during the study period is obvious, and the overall performance shows that the relatively weak impact areas are gradually expanding and the relatively strong role areas are contracting. Relatively weak areas are distributed in Jiangsu, Zhejiang, and Shanghai, with Shanghai and Chenzhou as the core clusters gradually expanding outward. With Hubei serving as the core cluster, relatively strong regions were distributed, and the operation was gradually reduced. This indicates that with the emergence of sustainable and green development concepts, the assessment of urban development benefits is no longer limited to socio-economic benefits. The social impact and environmental resources are simultaneously considered, and a considerable portion of the cities’ comprehensive benefits are increasing, while the scope and intensity of the benefits are gradually reduced.

During the study period, the spatial distribution of the resistance effect did not significantly vary. The main changes are represented by the expansion of the areas of medium and relatively strong levels, extending around the periphery, and the gradual transformation of relatively weak areas to relatively strong areas. It is clear that the relatively strong areas are all cities in economically developed regions, such as Jiangsu and Shanghai, while the relatively weak and medium areas are cities in economically slow-developing regions, such as Anhui, Hunan, and Hubei. This is primarily because rapid economic development results in high land utilization rates, a large proportion of construction land, a small proportion of ecological land, and a small total value of urban ecosystem services. As a result, resistance has a larger impact on the comprehensive and coordinated development of cities in this region and a smaller impact on regions such as Jiangxi and Anhui.

The change in the distribution of the impact role of recovery was not obvious, and the change in the range of each role intensity level was minimal. Due to the continuous improvement and optimization of land use structure, changes in the proportion of various land types affect each other. Although the change in the land use scale ratio within the regional scope is small, most central cities need to optimize and adjust the land structure layout, the level of urbanization and ecological resilience needs to be further improved, and the impact of urban resilience is gradually strengthened.

The variation in the distribution of adaptability mainly shows a shift from medium to relatively strong and strong grades and from relatively weak to medium grades. All grades generally showed an increasing trend, and the range of strong and medium grades increased significantly during the study period. Cities in the eastern part of the research region, including Jiangsu, Zhejiang, and Shanghai, are relatively strong areas that gradually expand with Shanghai as the center, while the relatively weak areas gradually shrink and are distributed in Jiangxi and Hunan provinces. With the implementation of urbanization, rural revitalization, and other strategies to further encourage better land use, adjustment and optimization of spatial layout, and rapid socioeconomic progress. However, in the interim, the fragmentation of the urban landscape intensified, and connectivity weakened. Owing to the large expansion of construction land, urban ecological land is fragmented and scattered, and landscape heterogeneity is further reduced. These phenomena are more common in economically developed regions, such as Shanghai, so the impact of adaptability on the cities in this region is obvious and gradually becomes stronger.

### Analysis of time series changes of core dimensions

#### Time series change of global core dimensions

**Figure 8:**
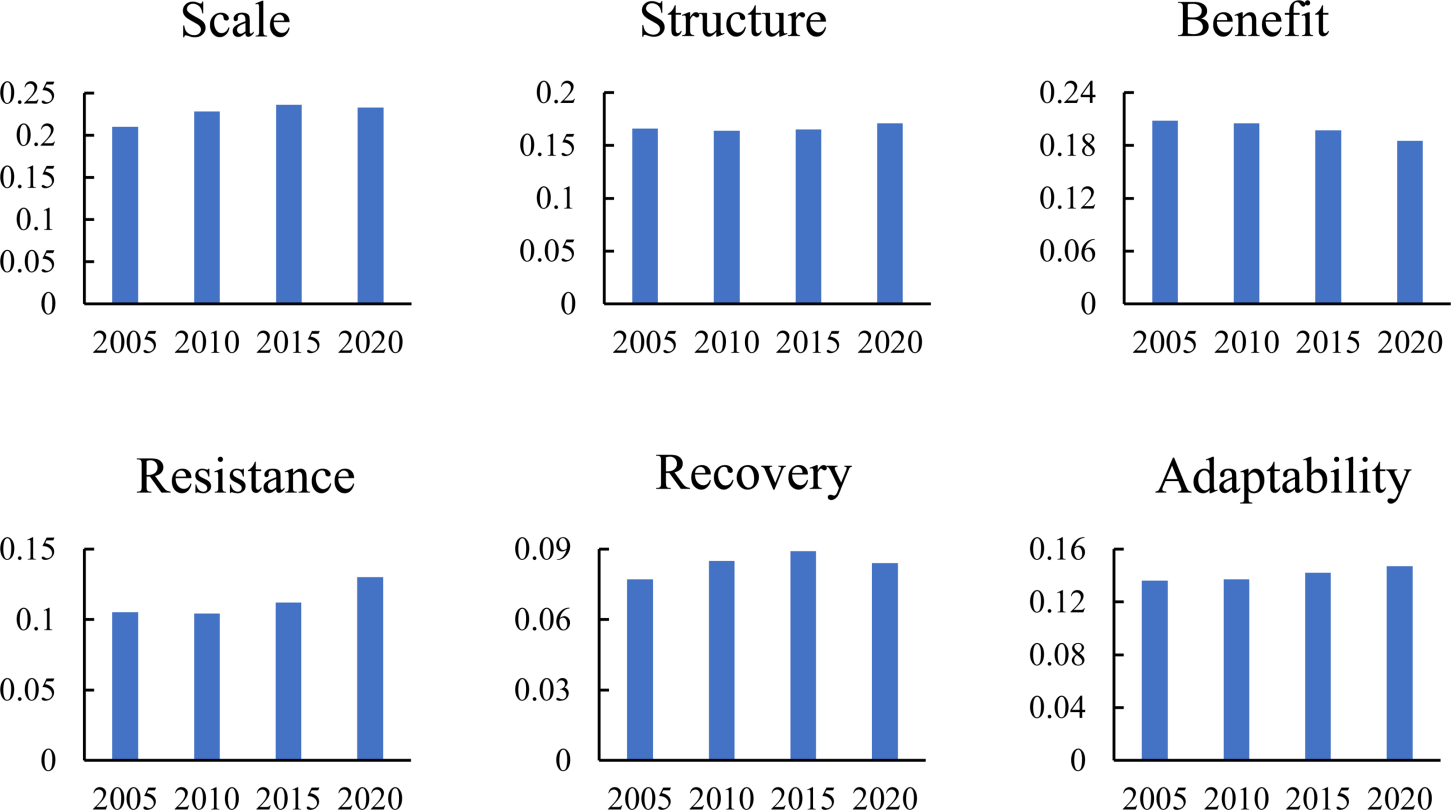
Average coefficient of each subsystem from 2005 to 2020.

The mean values of the coefficients for each dimension at each stage were calculated (Fig. 8) to identify the core dimensions at each stage. The scale dimension was always the primary factor influencing the CCD of urbanization and ecological resilience during the study period, as shown in the figure. Other dimensions have an impact on CCD, but the intensity of their impact changes. Structure, resistance, and adaptability show a positive relationship with coordination level, and the strength of their effects is generally on the rise. Recovery has an intrinsic correlation with coordination level, but its association is weak, and the difference is not obvious.

 According to the analysis of the average coefficients of each dimension, the core dimensions of the whole region are scale, efficiency, and structure, all of which are urbanization factors, and their effects are stronger than those of the ecological resilience dimensions. The level of urban economic growth is constantly correlated with the CCD between urbanization and ecological resilience. With the overall improvement of urbanization scale, structure, and efficiency, the layout of urban space, population, and economic structure is gradually becoming scientific and reasonable, which encourages the sustainable growth of urban society, economy, resources, and environment. The scale level is the most critical dimension affecting the CCD level in 2020, indicating that a reasonable expansion scale of cities is significant for comprehensive urban development.

### Identification of core dimensions of classified cities

**Figure 9:**
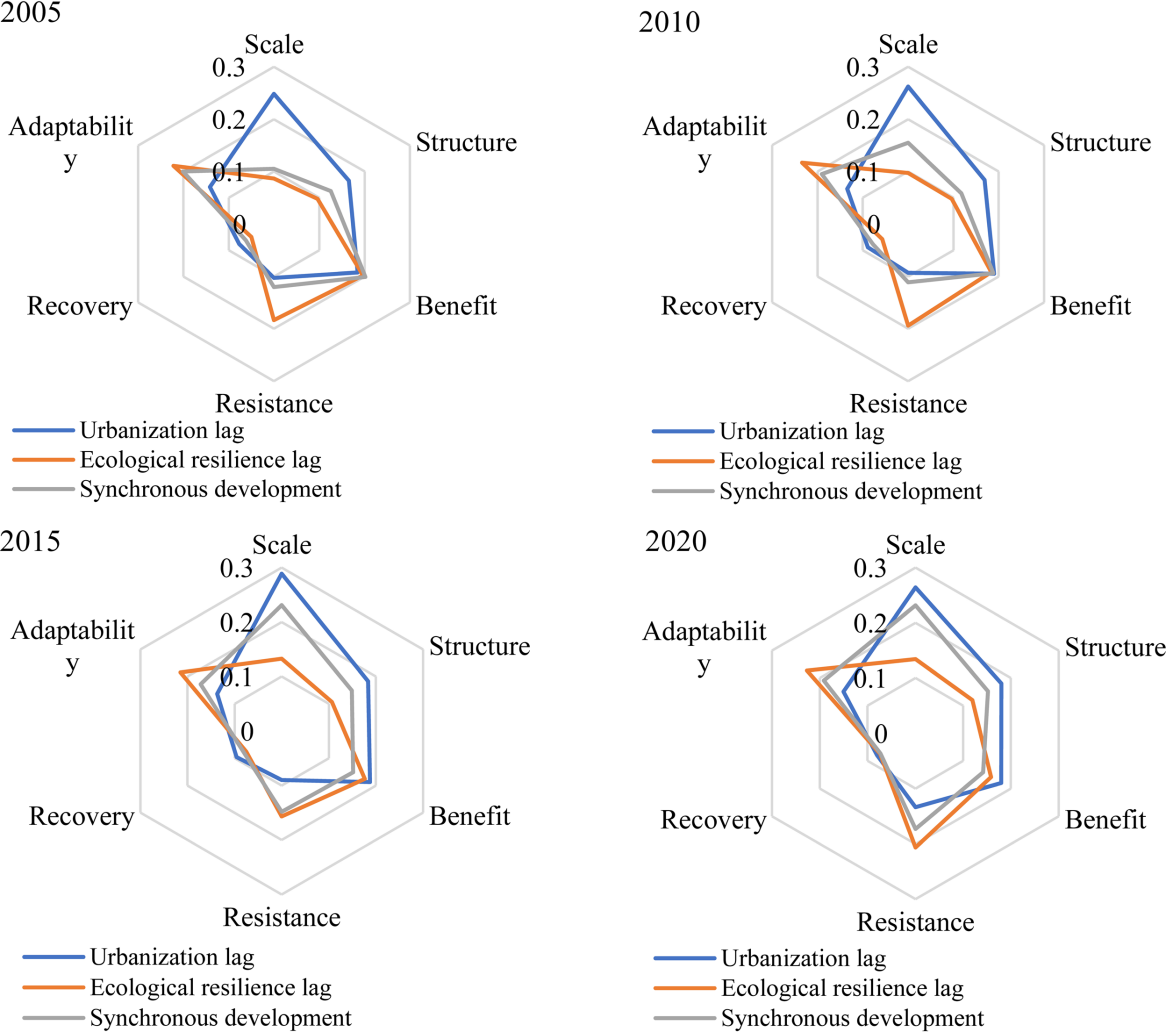
Identification of core dimensions of all types of cities, 2005–2020.

Identifying the core dimensions affecting the overall coupling coordination by type is the basis for realizing differentiated management of cities. To identify the core dimensions of cities with different types of characteristics, we calculated the mean values of the dimension coefficients for each type of city for four years: 2005, 2010, 2015, and 2020. We then integrated the average coefficients of the six dimensions from 2005 to 2020 to identify the three most influential dimensions, or the key core dimensions (Fig. 9).

 Scale, structure, and benefit are its three primary dimensions, which are primarily dispersed in places like Hunan, Hubei, Anhui, and Jiangxi. The area has a large scale of forest land, water areas, and other land types, the level of urban socio-economic development is the main factor restricting its coordinated development. Therefore, this type of city should follow the concept of sustainable development from the aspects of city size, structure, and benefit, abandon the traditional industrial type of heavy consumption and pollution, and develop tourism, green and innovative industries according to its own ecological advantages, to create a cultural tourism brand with local cultural characteristics, such as red Jinggang Mountain, ancient buildings Wudang Mountains, and ancient villages in southern Anhui, so as to enhance the influence and economic level of this region. Establishing reasonable urban development boundaries, optimizing land, industry, and population layout, fully considering the terrain constraints of Jiangxi’s hilly areas, and adopting a multi-center development model are conducive to habitat protection, reducing geological disaster risks, and promoting regional balanced development.

Benefit, resistance, and adaptability are the core dimensions of ecological resilience in lagging cities, among which adaptability has the strongest effect, followed by benefit and resistance. Urban ecology has suffered significant risks and losses as a result of large-scale construction and expansion in coastal areas, including Shanghai, Jiangsu, Zhejiang, and other major cities. As a result, cities in this region should implement ecological restoration, strengthen the development of green infrastructure (by creating urban green space or reducing construction intensity), encourage the optimization and upgrading of industrial structures, such as delimiting natural reserves, improving wildlife corridors, and ensuring the integrity of ecological patches like farmland and forest land, in order to increase the accessibility and affordability of their services.

Synchronous cities are mostly influenced by benefits, adaptability, and scale, with adaptability playing the most significant role. At present, synchronous development cities have been rid of low economic and ecological levels, and most of them are at the stage of balanced development of socioeconomic and ecological environment levels, paying attention to both socioeconomic development and proceeding to guarantee the level of ecosystem resilience, such as Changsha, Ningbo, Shaoxing, etc. The key to this type of city lies in the adaptability dimension. Compared to the structural elements of urbanization, adaptability is not only reflected in the reasonable layout of land use structure but also in the spatial distribution pattern of ecological landscape types it contains. For example, the expansion of urban disorderly road networks exacerbates landscape fragmentation and connectivity, and unreasonable resource utilization and urban construction disrupt the balance of ecosystem service supply and demand. Therefore, in the future, it is necessary to adjust the spatial layout of urban green space, implement ecological management zoning, develop green service industries, especially for traditional heavy industries, reduce carbon emissions and environmental pollution, and form a low-consumption, green, and low-carbon ecosystem service system.

## Discussion

### Reflections on the research of coupling relationship

Local ecologists have always been concerned with the coordinated growth of urbanization and ecological resilience. In short, it deals with how to divide up production and building tasks without endangering ecological sustainability and removing the barrier between resource consumption and economic growth. Urbanization and ecological resilience are thus treated equally in this research for coupling and coordinating analyses, examining their underlying mechanisms, and providing an approach for the formation of regional coordinated development plans.

First, as far as the CCD of urbanization and ecological resilience is concerned, research shows that most cities are in the basic coordination stage, yet never reached the most desirable development stage (*i.e.,* the superior balanced development stage) during the study period (Liu et al., 2018a). Further efforts should be made in the study area to achieve balanced development of urbanization and ecological resilience. The relative development types of urbanization and ecological resilience were dominated by lagging urbanization, with the number of cities as high as 65% and fewer cities with synchronized development (14%). This result shows that there is room for improvement in the urbanization level of most cities, such as urbanization scale, industrial structure, and economic benefits, which have become major factors in urban development (Fu et al., 2020; Yang, Wong Christina & Miao, 2021). Optimizing the structure and improving efficiency are rapidly becoming key issues in urban development.

Second, the overall level of CCD between urbanization and ecological resilience has continued to rise during the study period, with the level of urbanization continuously improving. The overall level of ecological resilience has remained relatively stable, while the central urban agglomeration has shown a significant downward trend (Xu et al., 2021; Yao et al., 2019), indicating that the relationship between urbanization and ecological resilience should be given equal attention. However, the overall growth rate was constrained and the pace of this transition was too slow. This is because the development level of each city varies, and the ecological environment varies from region to region. Urbanization and ecological resilience have their characteristics.

Finally, decision-makers must consider the spatial heterogeneity of the CCD between urbanization and ecological resilience. In the study area, each dimension presented a distinct spatial pattern with varying intensity. The complex spatial differences in the level of coupled coordination are related to the level of economic and social development, the urban-rural integration process, and regional natural conditions (Tan et al., 2017; Li, Lo & Zhang, 2020). The severity of the effect of different dimensions on CCD varies with city type. As a result, while developing suitable policy measures for a city, one should first take into account their actual circumstances before proposing specific remedies based on the key factors that influence the CCD of the city. Therefore, decision-makers can concentrate on the core dimensions and create efficient countermeasures that are appropriate for the local area, this article describes the essential characteristics of cities in various states.

### The significance of identifying the core dimensions of classified cities

The majority of the present drive studies remain at the time scale level and seldom examine the spatial influence of various components. However, the change in the coordination level of urbanization and ecological resilience coupling is a non-stationary spatiotemporal process, and changes in spatial location and time will cause changes in the relationship between factors. Therefore, this study comprehensively considers the spatial heterogeneity and temporal non-stationarity of the research object through the GTWR model, identifies and explains its driving effects, analyzes the impact factors under space–time dimensions, and analyzes the key driving dimensions according to the type of urban coordination. To more clearly reveal the driving mechanism of spatial differentiation of the urban coupling coordination level in the middle and lower reaches of the Yangtze River, and to realize the comprehensive optimization and improvement of the regional urban coupling coordination level, provide targeted suggestions for the future development of cities of different coordination types.

### Limitations and deficiencies

We also note that this study has certain limitations that need to be addressed in future research. First, the effect of human activities and related land development on various urban landscape types was the primary emphasis of the ecological resilience explored in this study. The selection of indicators related to the structure and scale of the ecological landscape is the main focus of this study within this research framework, and it carries out the research on the measurement of indicators in terms of urban resistance, adaptability, and recovery based on the situation of land type landscape, although it provides a convenient method for the assessment of urban ecosystem resilience. However, urban ecological resilience is comprehensively affected by many aspects. Using multi-source and field research data to conduct a comprehensive evaluation will help improve the accuracy of the assessment of ecosystem resilience. For instance, the percentage of land use data and the associated ecological elasticity coefficient are used in the ecological assessment model to calculate and measure resilience. It is required to collect multifarious and multi-channel risk data and perform a thorough evaluation based on the current restoration situation in the research region to properly consider the recovery of the ecosystem under pressure and risk. Future research efforts could concentrate on measuring resilience more precisely. On the other hand, this research analyzes the differences between each dimension in the role of CCD and proposes corresponding suggestions and strategies for different coordination types of cities. However, only six dimensions of urbanization and ecological resilience were selected in this study, and the impact of social aspects, such as land use change systems and cultural habits, were not considered. At the same time, it is worth noting that the construction of urban blue–green space is on the rise, which will alleviate pressure on the urban ecosystem. Because of this, in the future, thorough analysis of policy impact (Grain for Green, land reclamation), public preference, the proportion of green space, and other factors will aid in accurately and comprehensively exploring the underlying causes of coordinated development of urbanization and ecological resilience, to obtain more realistic and guiding conclusions. Sustainable urbanization development strategies are then formulated to better balance urbanization and ecological protection.

## Conclusions

This study analyzes the spatiotemporal changes and driving effects of the coupling coordination between urbanization and ecological resilience in the middle and lower reaches of the Yangtze River from the perspective of spatiotemporal heterogeneity by considering the urbanization process and ecological resilience characteristics of the study area. According to the research findings, there was a consistent upward trend in urbanization from 2005 to 2020, which is directly tied to the improvement of factors such as urban scale and benefit. Urbanization has decreased from the northeast to the center and western cities in space. The northeast has a relatively high level of urbanization and a pattern of ongoing growth; During the study period, the adaptive function of urban ecosystem has been weakening, and the level of ecological resilience has gradually declined, showing a spatial pattern of high in the south and low in the north. The main body of the coupling coordination level between urbanization and ecological resilience is in a basic coordination state, showing a continuous improvement trend. The relative development type is mainly characterized by lagging urbanization, with significant regional differences, which are closely related to the urban economic level and land landscape. The scale, connectivity, and variety of landscape types, as well as the degree of ecological resilience, decrease with increasing urbanization in economically developed places, and CCD also decreases. Based on the classification of the relative development level of urbanization and ecological resilience, the core driving factors are identified. The GTWR results show that the level of urbanization is the key to the coordinated development of cities, with the greatest impact on benefits, scale, and structure dimensions. The intensity of the urbanization dimension is significantly stronger than that of the ecological resilience dimension, with significant spatiotemporal differences.

This study assessed the quantitative evolution and driving role of the relationship between urbanization and ecological resilience in the middle and lower reaches of the Yangtze River. However, for other regions, the comprehensive indicator system and analytical model used in this study can be applied to other cities to assess the relationship between urbanization and urban ecological resilience. The results of this study may be useful for policymakers and researchers in other cities facing similar challenges related to sustainable urban development and environmental protection, and identifying the core influences on the coupled urbanization-ecological resilience relationship may help policymakers prioritize efforts to balance urbanization and ecological conservation. Therefore, the international generalizability of this study is high as it provides a useful framework and ideas for assessing the relationship between urbanization and ecological resilience in different cities.

##  Supplemental Information

10.7717/peerj.15869/supp-1 Supplemental Information 1 Comprehensive urbanization level evaluation system

10.7717/peerj.15869/supp-2 Supplemental Information 2 Ecological resilience evaluation index

10.7717/peerj.15869/supp-3 Supplemental Information 3 Comparison of indicator weights

10.7717/peerj.15869/supp-4 Supplemental Information 4 GTWR model parameters

## Additional Information and Declarations

### Data Availability

The following information was supplied regarding data availability: Data are available at Figshare: Xiao, Sheng (2022): Land use raster data.zip. figshare. Dataset. https://doi.org/10.6084/m9.figshare.21707603.v1. Xiao, Sheng (2022): China Urban Statistical Annual report.zip. figshare. Dataset. https://doi.org/10.6084/m9.figshare.21707606.v1. Xiao, Sheng (2022): Percentage of non-agricultural land of cities from 2005 to 2020. figshare. Dataset. https://doi.org/10.6084/m9.figshare.21731015.v3.
