# Supplementary material for: Research on the coupling coordination and driving role of urbanization and ecological resilience in the middle and lower reaches of the Yangtze River
Source: PeerJ. 2023 Sep 22;11:e15869. doi: 10.7717/peerj.15869 (PMC10519198; doi:10.7717/peerj.15869)
Supplement: Supplemental Information 1 [file peerj-11-15869-s001.docx]

| **First-level Indicators** | **Weight** | **Secondary indicators** | **Weight** | **Effect** | **Data source** |
| --- | --- | --- | --- | --- | --- |
| scale | 0.396 | The total industrial output value | 0.157 | + | The China Urban Statistical Yearbook of 2005–2020 (National bureau of statistics,  2005–2020). |
|  |  | Built-up area | 0.051 | + |  |
|  |  | Green space per capita | 0.057 | + |  |
|  |  | Unit Practitioners | 0.032 | + |  |
|  |  | Paved road surface per capita | 0.035 | + |  |
|  |  | Population density | 0.064 | + |  |
| Benefits | 0.467 | Gross regional product per capita | 0.135 | + |  |
|  |  | The average wage of employees | 0.035 | + |  |
|  |  | Total fixed asset investment | 0.084 | + |  |
|  |  | Industrial output per capita | 0.148 | + |  |
|  |  | Green coverage in built-up areas | 0.065 | + |  |
| Structure | 0.137 | The proportion of employees in secondary and tertiary industries | 0.013 | + |  |
|  |  | The proportion of urban construction land in the municipal area | 0.050 | + |  |
|  |  | The proportion of secondary and tertiary industries in GDP | 0.045 | + |  |
|  |  | The proportion of non-agricultural land | 0.029 | + |  |
