# Supplementary material for: Research on the coupling coordination and driving role of urbanization and ecological resilience in the middle and lower reaches of the Yangtze River
Source: PeerJ. 2023 Sep 22;11:e15869. doi: 10.7717/peerj.15869 (PMC10519198; doi:10.7717/peerj.15869)
Supplement: Supplemental Information 2 [file peerj-11-15869-s002.docx]

| **Indicators** | **Methodology and reference** | **Specific factors** | **Weights** | **Calculation process** | **Data source** |
| --- | --- | --- | --- | --- | --- |
| Resistance | Selecting the regulating ecosystem values such as climate regulation, gas regulation, Water Resources Conservation, soil formation and protection, and waste treatment from multi-media such as water, soil, and gas, which constitute the ecosystem resistance, and characterizes the strength of urban ecosystem resistance through ecosystem service values (Xie et al. 2005; Costanza et al. 1997). | ESV | 0.3705 | Calculation of ecosystem service values based on the "biomass factors of farmland ecosystems in different provinces of China" developed by Xie. | The 1000m×1000m resolution land use maps from the Resource and Environmental Science and Data Center and Earth System Science Data (2005-2020). |
| Adaptability | Taking the landscape structural stability of ecosystems to express their adaptability. Ecosystem structural stability is dependent on the stability of pattern organization in the landscape that is related to spatial heterogeneity and connectivity (Peterson et al. 2002; Turner et al. 1989; Peng et al. 2007). | Landscape pattern indices | 0.3309 | Landscape pattern indices such as the Shannon diversity index, area-weighted average patch fractal dimension, and landscape fragmentation were calculated by Fragstats 4.0. |  |
| Restoring | Ecosystem restoring, which can also be called ecological elasticity, indicates a system's capacity and potential to recover from a hazard and return to its original state (Peng et al. 2015; Yu et al. 2013). | Ecological elasticity | 0.2986 | Calculate the ecological elasticity using the coefficient and model for ecological elasticity suggested by Peng. |  |
