# Supplementary material for: Research on the coupling coordination and driving role of urbanization and ecological resilience in the middle and lower reaches of the Yangtze River
Source: PeerJ. 2023 Sep 22;11:e15869. doi: 10.7717/peerj.15869 (PMC10519198; doi:10.7717/peerj.15869)
Supplement: Supplemental Information 3 [file peerj-11-15869-s003.docx]

| **First-level Indicators** | **Secondary indicators** | **Weight-AHP** | **Weight-** **entropy** |
| --- | --- | --- | --- |
| scale | The total industrial output value | 0.157 | 0.114 |
|  | Built-up area | 0.051 | 0.073 |
|  | Green space per capita | 0.057 | 0.074 |
|  | Unit Practitioners | 0.032 | 0.082 |
|  | Paved road surface per capita | 0.035 | 0.029 |
|  | Population density | 0.064 | 0.039 |
| Benefits | Gross regional product per capita | 0.135 | 0.050 |
|  | The average wage of employees | 0.035 | 0.048 |
|  | Total fixed asset investment | 0.084 | 0.080 |
|  | Industrial output per capita | 0.148 | 0.299 |
|  | Green coverage in built-up areas | 0.065 | 0.005 |
| Structure | The proportion of employees in secondary and tertiary industries | 0.013 | 0.001 |
|  | The proportion of urban construction land in the municipal area | 0.050 | 0.053 |
|  | The proportion of secondary and tertiary industries in GDP | 0.045 | 0.006 |
|  | The proportion of non-agricultural land | 0.029 | 0.047 |
| Resistance | Ecosystem values (ESV) | 0.371 | 0.589 |
| Adaptability | Landscape Structural Stability of Ecosystems | 0.331 | 0.093 |
| Restoring | Ecological elasticity | 0.299 | 0.318 |
