# Supplementary material for: Research on the coupling coordination and driving role of urbanization and ecological resilience in the middle and lower reaches of the Yangtze River
Source: PeerJ. 2023 Sep 22;11:e15869. doi: 10.7717/peerj.15869 (PMC10519198; doi:10.7717/peerj.15869)
Supplement: Supplemental Information 4 [file peerj-11-15869-s004.docx]

| **Parameter** | **Minimum** | **Lower quartile** | **Median** | **Upper quartile** | **Maximum** |
| --- | --- | --- | --- | --- | --- |
| Intercept | 0.04 | 0.23 | 0.25 | 0.26 | 0.34 |
| Scale | 0.05 | 0.13 | 0.23 | 0.31 | 0.57 |
| Structure | 0.05 | 0.12 | 0.16 | 0.21 | 0.35 |
| Benefit | 0.07 | 0.16 | 0.2 | 0.24 | 0.35 |
| Resistance | -0.05 | 0.06 | 0.09 | 0.15 | 0.37 |
| Adaptability | -0.01 | 0.09 | 0.13 | 0.19 | 0.27 |
| Restoring | -0.01 | 0.06 | 0.08 | 0.1 | 0.23 |
| Diagnostic information | | |  |  |  |
| R^2^ | 0.9872 |  |  |  |  |
| R^2^Adjusted | 0.9869 |  |  |  |  |
| Sigma | 0.0088 |  |  |  |  |
| Residual Squares | 0.0239 |  |  |  |  |
| AICc | -1735.01 |  |  |  |  |
